# Dietary glycemic index, glycemic load and incidence of type 2 diabetes in Japanese men and women: the Japan public health center-based prospective study

**DOI:** 10.1186/1475-2891-12-165

**Published:** 2013-12-27

**Authors:** Shino Oba, Akiko Nanri, Kayo Kurotani, Atsushi Goto, Masayuki Kato, Tetsuya Mizoue, Mitsuhiko Noda, Manami Inoue, Shoichiro Tsugane

**Affiliations:** 1 Department of Health Promotion, National Institute of Public Health, 2-3-6 Minami, Wako, Saitama 351-0197, Japan; 2 Department of Epidemiology and Prevention, Clinical Research Center, National Center for Global Health and Medicine, Tokyo, Japan; 3 Department of Diabetes Research, National Center for Global Health and Medicine, Tokyo, Japan; 4 Department of Clinical Research, National Center for Global Health and Medicine, Tokyo, Japan; 5 Epidemiology and Prevention Division, Research Center for Cancer Prevention and Screening, National Cancer Center, Tokyo, Japan; 6 Graduate School of Medicine, the University of Tokyo, Tokyo, Japan

**Keywords:** Dietary glycemic index, Dietary glycemic load, Diabetes Mellitus, Cohort study, Japanese

## Abstract

**Background:**

Japanese diets contain a relatively high amount of carbohydrates, and its high dietary glycemic index and glycemic load may raise the risk of diabetes in the Japanese population. The current study evaluated the associations between the dietary glycemic index, glycemic load, and the risk of type 2 diabetes in a population based cohort in Japan.

**Methods:**

We observed 27,769 men and 36,864 women (45–75 y) who participated in the second survey of the Japan Public Health Center-based Prospective Study. The dietary glycemic index and glycemic load were estimated using a food-frequency questionnaire. The development of diabetes was reported in a questionnaire administered five years later, and the associations were analyzed using logistic regression after controlling for age, area, total energy intake, smoking status, family history of diabetes, physical activity, hypertension, BMI, alcohol intake, magnesium, calcium, dietary fiber and coffee intake, and occupation.

**Results:**

The dietary glycemic load was positively associated with the risk of diabetes among women: the multivariable-adjusted odds ratio comparing the highest vs. the lowest quartile was 1.52 (95% CI, 1.13-2.04; *P*-trend = 0.01). The association was implied to be stronger among women with BMI < 25 than the women with BMI ≥ 25. The dietary glycemic index was positively associated with the risk of diabetes among men with a high intake of total fat: the multivariable-adjusted odds ratio comparing the highest vs. the lowest quartile was 1.46 (95% CI, 0.94-2.28; *P*-trend = 0.04). Among women with a high total fat intake, those in the first and second quartiles of the dietary glycemic index had a significant reduced risk of diabetes, compared with those in the first quartile who had a lower total fat level (multivariable-adjusted odds ratio = 0.59 with 95% CI, 0.37-0.94, and odds ratio = 0.63 with 95% CI, 0.40-0.998 respectively).

**Conclusions:**

The population-based cohort study in Japan indicated that diets with a high dietary glycemic load increase the risk of type 2 diabetes among women. Total fat intake may modify the association between the dietary glycemic index and the risk of type 2 diabetes among men and women.

## Background

As carbohydrates are easily converted into glucose, and produce an insulin response generally [1], the consumption of food high in carbohydrates is naturally suspected as a risk factor of type 2 diabetes. However, previous prospective cohort studies have failed to find an association between total dietary carbohydrate and the risk of diabetes [2,3]. Japanese diets contain a relatively high amount of carbohydrates, with carbohydrates accounting for as much as 59% of the energy intake [4]. In a population-based prospective cohort study, we previously reported that white rice consumption was positively associated with the risk of diabetes among Japanese women [5]. White rice is the largest contributor to the dietary glycemic index (GI) and glycemic load (GL) in Japanese diets [6,7]. The dietary GI is the average GI value for an individual’s diet and represents the quality of carbohydrate invoking the postprandial glucose and insulin response, while the dietary GL represents both the quality and quantity of carbohydrate [8,9]. In addition to the effects of lower dietary fiber, vitamins, and minerals in white rice, a high dietary GI and GL produced by the regular consumption of white rice may raise the risk of diabetes in the Japanese population. In Japan, one prospective cohort study of male factory employees reported diets with high dietary GI increased risk of diabetes [10].

The associations between dietary GI and GL and the risk of diabetes were pronounced in individuals with a low cereal fiber intake [9,11,12]. Previous studies have also assessed the effects of modifiable risk factors of diabetes, including obesity and low physical activity, on these associations [10,12-15]. In addition to these factors, several experimental studies have implied that the total fat intake might also impact the associations [16-18], although not all studies support this view [19]. The aim of the current study was to assess the associations between dietary GI and GL and the risk of type 2 diabetes among Japanese men and women in a large-scale population-based cohort study. We further conducted a stratified analysis according to BMI, physical activity, and total fat and dietary fiber intake to seek suggestions to people with the modifiable risk factors.

## Materials and methods

### Study population

The Japan Public Health Center-based Prospective Study was launched in 1990 for Cohort I, and Cohort II was added in 1993. The study design has been previously described [20]. Briefly, subjects were from eleven public health center areas across Japan. All the subjects were residents of the respective areas and were 40–69 years old at the time of the baseline survey. A questionnaire was administered at baseline, and the participants were informed of the objectives of the study at that time. Subjects who responded to the questionnaire were regarded as having consented to participate in the study. Follow-up surveys were conducted at 5 and 10 years after the baseline survey. Information on medical histories and health-related lifestyle factors was obtained from the questionnaire administered at each survey. As the 5-year survey included more comprehensive information on food intake frequency than the baseline survey, it was used as the starting point to identify the development of diabetes. This study was approved by the Institutional Review Board of the National Cancer Center, Tokyo, Japan.

There were 140,160 eligible subjects, and 113,403 of them responded to the questionnaire at the time of the baseline survey, yielding a response rate of 80.9%. Among the subjects who responded at the baseline survey, 77,540 (68.4%) responded to both the 5-year and 10-year follow-up surveys. Those who reported that they had been diagnosed as having diabetes or who reported a history of cancer, cerebrovascular disease, myocardial infarction, chronic liver disease, or renal disease at the baseline or 5-year survey were excluded from the analysis (n = 11,732). Furthermore, subjects whose dietary information was missing or whose total energy intake lay outside the mean ± 3 standard deviations according to sex were also excluded (n = 1,175). After these exclusions, the analysis included 64,633 eligible participants, consisting of 27,769 men and 36,864 women.

### Dietary assessment

At the 5-year survey, food frequency questionnaire (FFQ) containing 147 items was administered to assess the habitual diets over the past year [21]. The validity and reproducibility of the FFQ has been previously assessed [22,23]. The Spearman rank correlation coefficients for energy adjusted carbohydrate intake measured using the 28-day diet records and using the FFQ were 0.66 for men and 0.45 for women in a subsample of cohort I, and 0.69 for men and 0.47 for women in a subsample of cohort II [23]. The correlations between the daily intake of rice measured in the FFQ and the dietary records were examined in a subsample of cohort I and cohort II and were 0.67 for men and 0.54 for women [24].

The GI values for single food items on the FFQ were derived based on available publications [25,26]. Whenever there was more than one value for an item, the GI values were averaged, with preference given to data from studies of subjects with normal glucose tolerance, and Japanese studies. The carbohydrate intake after subtracting the dietary fiber intake, regarded as the available/digestible carbohydrate intake, was used to calculate the dietary GI and dietary GL. This protocol follows a proposal for testing the GI value for individual food items based on the carbohydrate content available for absorption in the small intestine [27]. Previous studies set the cutoff for excluding food items as 3.5 g for the total or available carbohydrate per serving [26,28]. In our FFQ, *Sake* (Japanese rice wine), which contained 8.8 g of available carbohydrate per serving, was assigned the same GI as beer (GI = 66), as no GI value was available. GI values were also not available for four kinds of vegetables containing more than 3.5 g/serving of total carbohydrate: bitter gourd, chard, and loofah (which contain very small amounts of available carbohydrate) as well as onion (which contains 3.42 g of available carbohydrate per serving). As the GI values were also not available for similar vegetables in the same family and it is unlikely that they would induce a significant rise in blood glucose [27], we assigned them a value of 0. The dietary GI was computed by summing the products of the available carbohydrate content per serving for each food item, multiplied by the number of servings of food per day, multiplied by its glycemic index, and then dividing this sum by the total daily intake of available carbohydrate [8]. Dietary GL was computed as the dietary GI but was divided by 100 instead of the total available carbohydrate intake [9].

### Development of diabetes

The development of newly diagnosed diabetes for 5 years, between the 5-year survey and the 10-year survey, was identified using the questionnaire. The validity of diabetes reported in the questionnaire was previously assessed for study participants in three districts of the study areas. Based on their medical records, 94% of the participants who reported a diagnosis of diabetes were confirmed to have diabetes among those whose medical records were available [29].

### Statistical analysis

All the analyses were performed for men and women separately, as the estimated prevalence of diabetes varies by sex in Japan [30]. The characteristics of the participants were presented according to the quartile of dietary GI. A logistic regression analysis was utilized to assess the association between dietary GI/GL and the risk of diabetes. The odds ratios for the incidence of diabetes and 95% CI were calculated for quartiles of each dietary measure relative to the lowest quartile.

For each dietary variable, we assessed the risk using two models: one adjusted for age and study area, and the other adjusted for age, study area and potential confounding factors including log-transformed total energy intake, smoking status (never, current, former, missing), family history of diabetes (ever, never), physical activity (quartiles, missing), hypertension (ever, never), BMI (14 - <21, 21 - <23, 23 - <25, 25 - <27, 27 - <40, missing/outlier), intake of alcohol (men: nondrinker, occasional drinker, drinker with a consumption of <150, 150 - <300, 300 - <450, ≥450 g ethanol/week, missing. women: nondrinker, occasional drinker, <150, ≥150 g ethanol/week, missing), energy adjusted intake of log-transformed magnesium, calcium, and dietary fiber, coffee consumption (almost never, <1, 1, ≥2 cups/day), and occupation (agriculture/forestry/fishery, salaried/self-employed/professional, housework/unemployed/retired, missing). Metabolic equivalents per day was obtained to measure the level of physical activity from a questionnaire administered in the 5-year survey, and this measure has been previously validated [31]. To test for linear trends across categories, we modeled the median of each quartile category of dietary measure as a continuous variable. The dietary GL and nutrients intake in the model were adjusted for the total energy intake using a residual model [32]. Stratified analyses were conducted in a multivariable model to evaluate modifying effect, whether the association between each dietary measure and the risk of diabetes varied according to the BMI, the level of physical activity, or the intake of total fat and dietary fiber. The cut-off point chosen for BMI was 25 and that for other variables was median value. All the statistical analyses were conducted using SAS software (SAS Institute Inc., Cary, NC).

## Results

Table 1 summarizes the characteristic of the participants according to the quartile distribution of the dietary GI. The dietary GI was positively associated with age. Participants in the lowest dietary GI quartile were more likely to be current smokers. Participants with a low dietary GL were more likely to consume large amounts of alcohol and to be current smokers (data not shown).

**Table 1:** Characteristics of study participants by quartile of dietary glycemic index in the JPHC Study

	**Quartile dietary glycemic index**							
	**1**		**2**		**3**		**4**	
*Men (n = 27769)*								
Age,mean (SD)	55.5	(7.8)	56.3	(7.8)	56.6	(7.8)	57.5	(7.7)
Physical activity, metabolic equivalents -h/d, mean (SD)^a^	33.2	(6.7)	33.8	(6.7)	34.3	(6.8)	34.5	(6.8)
BMI, mean (SD)^b^	23.7	(2.9)	23.6	(2.8)	23.5	(2.8)	23.4	(2.8)
History of hypertension, %	14.3		16.8		18.0		18.7	
Current smokers^c^, %	51.5		45.2		44.3		44.6	
Occupation^d^, %								
Agriculture/forestry/fishery	21.6		27.6		31.6		37.4	
Salaried/self-employed/professional	65.0		59.4		54.9		49.9	
Housework/unemployed/retired	13.4		13.0		13.5		12.8	
Dietary GI, mean (SD)	55	(4)	61	(1)	64	(1)	68	(2)
Dietary GL, mean (SD)	159	(59)	171	(50)	175	(51)	180	(56)
Total energy, kcal/d, mean (SD)	2460	(876)	2309	(699)	2174	(656)	1991	(626)
Carbohydrate, g/d, mean (SD)	302	(112)	294	(86)	285	(84)	274	(85)
Rice, g/d, mean (SD)	321.4	(154.5)	410.9	(151.7)	470.6	(170.5)	545.7	(199.5)
Rice with millet or barley, g/d, mean (SD)	24.2	(77.7)	26.7	(88.2)	25.2	(91.6)	20.7	(83.3)
Bread, g/d, mean (SD)	27.0	(34.7)	25.2	(36.2)	20.7	(35.7)	17.3	(51.0)
Udon, g/d, mean (SD)	73.9	(109.7)	58.4	(67.5)	45.1	(48.4)	29.1	(33.3)
Magnesium intake, mg/d, mean (SD)	349.4	(146.6)	312.7	(109.1)	277.7	(97.6)	231.6	(84.0)
Calcium, mg/d, mean (SD)	737	(497)	574	(284)	469	(220)	350	(177)
Dietary fiber, g/d, mean (SD)	15	(8)	13	(6)	12	(6)	9	(4)
Total fat, g/d, mean (SD)	75.2	(39.1)	64.4	(29.5)	55.0	(26.4)	42.3	(22.9)
Alcohol, g/week, (SD)	197.5	(241.9)	200.8	(235.8)	214.7	(243.2)	230.8	(249.3)
Coffee intake, almost never^e^, %	15.8		22.8		32.1		51.7	
*Women (n = 36864)*								
Age,mean (SD)	55.3	(7.6)	56.1	(7.6)	57.2	(7.8)	58.9	(8.0)
Physical activity, metabolic equivalents -h/d, mean (SD)^a^	32.8	(5.7)	32.9	(5.7)	32.9	(5.8)	32.7	(5.8)
BMI, mean (SD)^b^	23.3	(3.0)	23.4	(3.0)	23.5	(3.1)	23.6	(3.2)
History of hypertension, %	15.1		16.8		19.2		22.9	
Current smokers^c^, %	6.5		4.4		3.8		4.1	
Occupation^d^, %								
Agriculture/forestry/fishery	16.2		22.8		27.4		32.2	
Salaried/self-employed/professional	42.5		37.0		32.8		27.8	
Housework/unemployed/retired	41.3		40.2		39.7		40.0	
Dietary GI, mean (SD)	54	(3)	59	(1)	62	(1)	67	(2)
Dietary GL, mean (SD)	143	(53)	154	(43)	151	(39)	148	(44)
Total energy, kcal/d, mean (SD)	2215	(793)	2040	(598)	1835	(508)	1565	(509)
Carbohydrate, g/d, mean (SD)	283	(103)	276	(77)	256	(66)	232	(70)
Rice, g/d, mean (SD)	256.4	(126.1)	334.0	(117.8)	371.1	(120.7)	419.4	(142.8)
Rice with millet or barley, g/d, mean (SD)	24.8	(71.2)	27.6	(79.9)	27.1	(81.2)	21.6	(70.7)
Bread, g/d, mean (SD)	32.4	(32.8)	31.9	(39.8)	27.8	(43.5)	24.6	(59.1)
Udon, g/d, mean (SD)	62.5	(89.7)	52.0	(54.3)	41.0	(43.7)	27.1	(31.7)
Magnesium, mg/d, mean (SD)	355.8	(145.7)	312.2	(105.9)	269.8	(86.1)	211.2	(79.0)
Calcium, mg/d, mean (SD)	852	(530)	641	(280)	516	(219)	362	(181)
Dietary fiber, g/d, mean (SD)	18	(9)	16	(7)	13	(6)	10	(5)
Total fat, g/d, mean (SD)	78.5	(37.0)	66.0	(28.9)	55.6	(24.4)	41.8	(23.2)
Alcohol, g/week, (SD)	18.1	(69.0)	14.1	(62.4)	13.2	(63.3)	11.2	(56.8)
Coffee intake, almost never^e^, %	13.4		20.8		30.3		47.2	

^a^n = 3117 for men and n = 30429 for women.

^b^n = 27205 for men n = 35972 for women.

^c^n = 27131 for men n = 34536 for women.

^d^n = 26876 for men, n = 35597 for women.

^|e^n = 26325 for men, n = 34986 for women.

Table 2 summarizes the associations between dietary GI and GL and the incidence of diabetes. Not a dose–response relationship between the dietary GI and the risk of diabetes, but a non-linear relationship was observed among men; the odds ratio increased significantly to 1.29 in the third quartile but decreased to a non-significant value in the fourth quartile. A similar risk pattern was observed across the quartiles for dietary GL among men. The dietary GI was significantly associated with the risk of diabetes among women in the analysis adjusted for age and study area, but the association was attenuated and no longer significant after additional adjustments for multiple factors. The dietary GL was significantly associated with an increased risk of diabetes among women.

**Table 2:** Odds ratio of diabetes according to quartile of dietary GI/GL in the JPHC Study

	**Quartile**							***P*-trend**
	**1**	**2**		**3**		**4**		
*Men* (n = 27769)								
Dietary GI								
No. of cases	152		172		187		179	
Age and area adjusted OR (95% CI)	1.00	1.13	(0.91-1.41)	1.24	(0.99-1.54)	1.17	(0.94-1.46)	0.12
Multivariate OR^a^ (95% CI)	1.00	1.17	(0.93-1.48)	1.29	(1.01-1.65)	1.19	(0.90-1.59)	0.15
Energy-adjusted dietary GL								
No. of cases	147		194		184		165	
Age and area adjusted OR (95% CI)	1.00	1.34	(1.08-1.67)	1.27	(1.02-1.59)	1.13	(0.90-1.43)	0.46
Multivariate OR (95% CI)	1.00	1.34	(1.07-1.69)	1.27	(0.998-1.62)	1.16	(0.89-1.53)	0.40
*Women* (n = 36864)								
Dietary GI								
No. of cases	105		118		124		153	
Age and area adjusted OR (95% CI)	1.00	1.10	(0.85-1.44)	1.12	(0.86-1.46)	1.34	(1.03-1.72)	0.03
Multivariate OR (95% CI)	1.00	1.05	(0.79-1.38)	1.03	(0.77-1.38)	1.14	(0.81-1.60)	0.51
Energy-adjusted dietary GL								
No. of cases	92		124		126		158	
Age and area adjusted OR (95% CI)	1.00	1.35	(1.02-1.77)	1.34	(1.02-1.76)	1.66	(1.28-2.17)	0.0003
Multivariate OR (95% CI)	1.00	1.32	(0.999-1.75)	1.28	(0.96-1.71)	1.52	(1.13-2.04)	0.01

^a^Age, public health center area, total energy intake, smoking status, family history of diabetes, physical activity (metabolic equivalents), hypertension, BMI, alcohol intake, magnesium, calcium, dietary fiber and coffee intake and occupation.

Table 3 summarizes the association between dietary GI and the risk of diabetes stratified according to the risk factors of diabetes. Dietary GI was significantly associated with diabetes among men with a high intake of total fat. Associations were also suggested among women with a BMI of less than 25, a high level of physical activity, and a high intake of total fat, although they were not statistically significant. Stratified analysis did not show a clear difference of the association by level of dietary fiber intake.

**Table 3:** **Odds ratios**^**a **^**of diabetes according to quartiles of dietary GI, stratified analysis, the JPHC Study**

	**Quartile of dietary glycemic index**							***P*-trend**
	**1**	**2**		**3**		**4**		
**Men (n)**	6943		6942		6942		6942	
**BMI**^**b,c**^								
BMI I < 25, OR (95% CI)	1.00	1.18	(0.85-1.62)	1.15	(0.82-1.62)	1.13	(0.76-1.68)	0.55
BMI I ≥ 25, OR (95% CI)	1.00	1.23	(0.87-1.73)	1.50	(1.05-2.14)	1.29	(0.85-1.98)	0.13
**Physical activity** (metabolic equivalents **-h/d**)^b,d^								
Lower than median, OR (95% CI)	1.00	0.96	(0.68-1.37)	1.24	(0.86-1.80)	1.14	(0.73-1.77)	0.39
Median or higher, OR (95% CI)	1.00	1.52	(1.06-2.19)	1.32	(0.89-1.97)	1.29	(0.81-2.05)	0.34
**Dietary fiber intake**^**e**^								
Lower than median, OR (95% CI)	1.00	1.32	(0.93-1.89)	1.23	(0.86-1.78)	1.15	(0.78-1.71)	0.65
Median or higher, OR (95% CI)	1.00	1.07	(0.78-1.47)	1.36	(0.97-1.92)	1.26	(0.81-1.95)	0.14
**Total fat intake**								
Lower than median, OR (95% CI)	1.00	1.01	(0.69-1.47)	1.16	(0.80-1.67)	1.05	(0.71-1.56)	0.73
Median or higher, OR (95% CI)	1.00	1.31	(0.97-1.78)	1.45	(1.03-2.06)	1.46	(0.94-2.28)	0.04
**Women (n)**	9216		9216		9216		9216	
**BMI**^**b,c**^								
BMI I < 25, OR (95% CI)	1.00	0.91	(0.59-1.39)	1.23	(0.80-1.88)	1.24	(0.75-2.05)	0.28
BMI I ≥ 25, OR (95% CI)	1.00	1.23	(0.85-1.79)	0.88	(0.58-1.34)	1.02	(0.64-1.65)	0.79
**Physical activity** (metabolic equivalents **-h/d**)^b,d^								
Lower than median, OR (95% CI)	1.00	0.88	(0.59-1.30)	0.88	(0.58-1.35)	0.71	(0.42-1.20)	0.25
Median or higher, OR (95% CI)	1.00	1.49	(0.92-2.43)	1.46	(0.86-2.50)	1.69	(0.91-3.15)	0.12
**Dietary fiber intake**^**e**^								
Lower than median, OR (95% CI)	1.00	1.26	(0.76-2.07)	1.16	(0.71-1.90)	1.29	(0.77-2.16)	0.41
Median or higher, OR (95% CI)	1.00	0.94	(0.67-1.33)	0.96	(0.65-1.42)	1.11	(0.68-1.80)	0.81
**Total fat intake**								
Lower than median, OR (95% CI)	1.00	0.93	(0.60-1.44)	0.78	(0.50-1.21)	0.80	(0.51-1.28)	0.32
Median or higher, OR (95% CI)	1.00	1.09	(0.76-1.58)	1.19	(0.78-1.82)	1.62	(0.96-2.72)	0.10

^a^Adjusted for age, public health center area, total energy intake, smoking status, family history of diabetes, physical activity (metabolic equivalents), hypertension, BMI, alcohol intake, magnesium, calcium, dietary fiber and coffee intake and occupation.

^b^Only individuals whose data was available were analyzed. BMI: 27,205 for men and 35,972 for women. Physical activity: 23117 for men and 30429 for women.

^c^The model was not adjusted for BMI.

^d^The model was not adjusted for physical activity.

^e^The model was not adjusted for dietary fiber intake.

Table 4 summarizes the association between dietary GL and the risk of diabetes stratified according to the same factors mentioned above. Among men whose total fat intake was higher than the median, the odds ratio significantly increased to 1.51 in the second quartile and 1.46 in the third quartile, but no significant increase was observed in the fourth quartile. A significantly positive association between the dietary GL and the risk of diabetes was observed among women whose BMI was less than 25. There was no clear difference of the association by the level of dietary fiber intake.

**Table 4:** **Odds ratios**^**a **^**of diabetes according to quartiles of dietary GL, stratified analysis, JPHC Study**

	**Quartile of energy adjusted dietary glycemic load**							***P*-trend**
	**1**	**2**		**3**		**4**		
**Men (n)**	6943		6942		6942		6942	
**BMI**^**b,c**^								
BMI I < 25, OR (95% CI)	1.00	1.09	(0.80-1.50)	1.17	(0.84-1.62)	1.05	(0.72-1.52)	0.77
BMI I ≥ 25, OR (95% CI)	1.00	1.05	(0.72-1.52)	1.39	(0.97-2.00)	1.31	(0.88-1.96)	0.34
**Physical activity** (metabolic equivalents **-h/d**)^b,d^								
Lower than median, OR (95% CI)	1.00	1.51	(1.06-2.14)	1.30	(0.89-1.91)	1.26	(0.81-1.94)	0.46
Median or higher, OR (95% CI)	1.00	1.30	(0.89-1.89)	1.57	(1.07-2.31)	1.30	(0.85-1.99)	0.21
**Dietary fiber intake**^**e**^								
Lower than median, OR (95% CI)	1.00	1.25	(0.91-1.72)	1.21	(0.86-1.69)	1.20	(0.82-1.76)	0.43
Median or higher, OR (95% CI)	1.00	1.45	(1.04-2.03)	1.34	(0.94-1.91)	1.09	(0.74-1.61)	0.81
**Total fat intake**								
Lower than median, OR (95% CI)	1.00	1.19	(0.81-1.74)	1.12	(0.75-1.68)	1.10	(0.70-1.74)	0.88
Median or higher, OR (95% CI)	1.00	1.51	(1.12-2.03)	1.46	(1.04-2.06)	1.25	(0.84-1.86)	0.17
**Women (n)**	9216		9216		9216		9216	
**BMI**^**b,c**^								
BMI I < 25, OR (95% CI)	1.00	1.37	(0.89-2.12)	1.29	(0.80-2.08)	1.26	(0.70-2.29)	0.03
BMI I ≥ 25, OR (95% CI)	1.00	1.19	(0.79-1.77)	1.01	(0.64-1.59)	1.22	(0.69-2.16)	0.09
**Physical activity** (metabolic equivalents **-h/d**)^b,d^								
Lower than median, OR (95% CI)	1.00	1.13	(0.74-1.73)	1.01	(0.63-1.63)	0.91	(0.49-1.70)	0.57
Median or higher, OR (95% CI)	1.00	1.41	(0.86-2.32)	1.05	(0.58-1.90)	1.12	(0.52-2.43)	0.48
**Dietary fiber intake**^**e**^								
Lower than median, OR (95% CI)	1.00	1.51	(1.002-2.28)	1.38	(0.90-2.11)	1.56	(0.98-2.49)	0.11
Median or higher, OR (95% CI)	1.00	1.13	(0.77-1.66)	1.15	(0.78-1.70)	1.37	(0.93-2.02)	0.12
**Total fat intake**								
Lower than median, OR (95% CI)	1.00	1.09	(0.68-1.77)	1.13	(0.70-1.82)	1.32	(0.79-2.20)	0.23
Median or higher, OR (95% CI)	1.00	1.37	(0.95-1.96)	1.14	(0.75-1.75)	1.44	(0.93-2.23)	0.14

^a^Age, public health center area, total energy intake, smoking status, family history of diabetes, physical activity (metabolic equivalents), hypertension, BMI, alcohol intake, magnesium, calcium, dietary fiber and coffee intake and occupation.

^b^Only individuals whose data was available were analyzed. BMI: 27,205 for men and 35,972 for women. Physical activity: 23117 for men and 30429 for women.

^c^The model was not adjusted for BMI.

^d^The model was not adjusted for physical activity.

^e^The model was not adjusted for dietary fiber intake.

**Figure 1:**
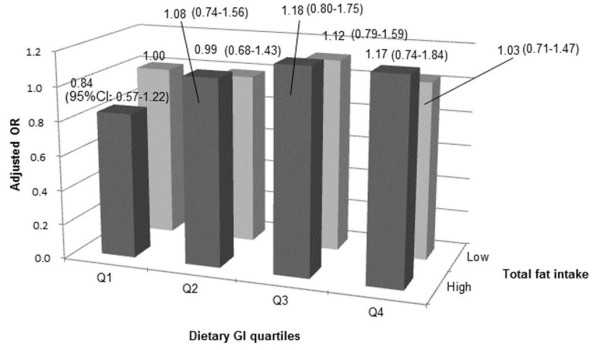
**Adjusted odds**^**1 **^**ratio of development of diabetes by different levels of total fat intake and dietary glycemic index among men in the Japan Public Health Center-based Prospective Study.** 1 Adjusted for age, public health center area, total energy intake, smoking status, family history of diabetes, physical activity (metabolic equivalents), hypertension, BMI, alcohol intake, magnesium, calcium, dietary fiber and coffee intake and occupation.

**Figure 2:**
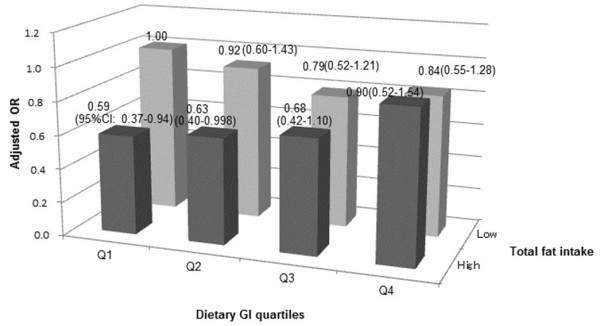
**Adjusted odds**^**1 **^**ratio of development of diabetes by different levels of total fat intake and dietary glycemic index among women in the Japan Public Health Center-based Prospective Study.** 1 Adjusted for age, public health center area, total energy intake, smoking status, family history of diabetes, physical activity (metabolic equivalents), hypertension, BMI, alcohol intake, magnesium, calcium, dietary fiber and coffee intake and occupation.

Since we observed a significant association between dietary GI and the risk of diabetes when stratified according to the total fat intake among men as well as a similar, but non-significant, association among women, we conducted an post-hoc analysis to assess the joint effect of dietary GI and total fat intake on the risk of diabetes by cross-classifying participants according to these variables (Figures 1 and 2). Among women, those in the first and second quartiles of dietary GI with a high total fat intake had a significantly lower risk of diabetes, compared with women in the first quartile of the dietary GI with a lower total fat intake. Among women, a positive association between the dietary GI and the risk of diabetes was observed only for those with a high total fat intake.

## Discussion

In this large scale population based cohort study, a higher dietary GL was associated with an increased risk of diabetes among women. The dietary GI was also implied to be associated with the risk of diabetes among women, but the association was attenuated and no longer significant after controlling for potential risk factors of diabetes. This is the prospective study conducted among men and among women in the general Japanese population.

A recent study among Japanese male workers reported that the dietary GI was significantly associated with an increased risk of diabetes [10], but a similar association was not observed in the current study. The different characteristics of the participants might explain the difference in these results, as the current study was conducted among the general population. The cohort in the previous study consisted of employees at a factory, whereas the current study was based on the male and female population in Japan.

The dietary GL was positively associated with the risk of diabetes among women in the current study. A few studies in Europe have even observed a protective effect of dietary GL on the risk of diabetes in certain models [33,34], but a positive association between dietary GL and the risk of diabetes has been reported in meta-analyses [35,36]. Among the women in the present cohort, a positive association between rice intake and the risk of diabetes was observed [5]. Considering that rice is a high GI food and that it is the major source of carbohydrates in Japanese diets, the current result seems reasonable, as the dietary GL reveals both the quantity and quality of carbohydrates in diets. A cohort study in Chinese women also reported a positive association between the dietary GL and the risk of diabetes [14], and a recent cross-sectional study of obese males and females in Japan reported an association between the dietary GL and the HbA1c level [37]. No association was observed between the dietary GL and the risk of diabetes among men. Although only two studies have been previously conducted in men alone [10,11], they also failed to find an association. A low dietary GL was associated with a current smoking status and a high alcohol intake in the current study in both men and women, but the degree of exposure to smoking and heavy drinking was higher among men than among women. Although we carefully controlled for these variables, the potential for residual confounders related to unhealthy lifestyle habits may not be ruled out.

The stratified analysis shows that women whose BMI was less than 25 kg/m^2^ had a positive association between dietary GL and the risk of diabetes, whereas the association was weaker among those with a higher BMI. The association with dietary GI was also implied in the same group, albeit non-significantly. Several studies have reported an increased risk of diabetes in individuals with a higher dietary GI or GL among subjects with a high BMI [12-14]. Only a few studies have reported a positive association between the risk of diabetes and the dietary GI among subjects with a low BMI [10,15], and no study has reported an association between dietary GL and the risk of diabetes among subjects with a low BMI. However, the association between the dietary GI and the risk of diabetes was reportedly pronounced in individuals with a low level of insulin resistance in a study of Japanese male workers [10]. As insulin resistance is less frequently observed among people with low BMI, this finding showed similarity as current results. There is a possibility that a high dietary GL affects women with decreased beta-cell function. An inverse association has been observed between BMI and insulin secretion among subjects with normal glucose tolerance and among patients with diabetes who have good glycemic control [38,39]. The effect of physical activity on the association with the dietary GI, which was implied among women in the current study, was also inconsistent in previous studies [12-14].

A positive association between the dietary GI and the risk of diabetes in both men and women was suggested among individuals with a high level of total fat intake, but not among those with a lower level of total fat intake. Although no previous epidemiological studies have assessed the effect of dietary fat intake on the association between the dietary GI and the risk of diabetes, the findings from human experimental studies have indicated that the consumption of potato, one of the highest GI food items, together with margarine, butter, or corn oil reduced the blood glucose response, compared with the consumption of potato by itself [16-18]. A high total fat intake did not lower the risk of diabetes among those with a high dietary GI in the current study. While the exact reason is unclear, a threshold dietary GI for the risk of diabetes may exist among individuals who regularly consume high levels of total fat.

The current study has several limitations. The development of diabetes was determined using a self-administered questionnaire, the FFQ, which was not specifically designed to estimate the dietary GI or GL. Nevertheless, such FFQ has been utilized in several epidemiological cohort studies [9-11,14,40]. The correlation between carbohydrate intake measured using the FFQ and the food record was relatively high, and the correlation between the two measures for the intake of rice, which was the major source of carbohydrates in the current population, was also high.

## Conclusion

The current population-based cohort study in Japan observed an association between the dietary GL and the risk of type 2 diabetes among women. The dietary GI was also associated with the risk of type 2 diabetes among men whose total fat intake was higher than the median level. Further studies are needed to assess the association between dietary GI, GL and the risk of type 2 diabetes in populations with different habitual diets.

## Abbreviations

GI: Glycemic index; GL: Glycemic load; FFQ: Food frequency questionnaire.

## Competing interests

The authors declare that they have no competing interests.

## Authors’ contributions

ST, MI, TM and MN were responsible for the study conception and the design of the study. ST and MI acquired data. SO, AN and KK performed the analysis. SO, AN, KK, AG, MK, TM and MN made contributions to interpretation of data. SO drafted the manuscript. All authors revised the manuscript critically for important intellectual content. All authors read and approved the final manuscript.
